# Interactions between Medical Residents and Drug Companies: A National Survey after the Mediator® Affair

**DOI:** 10.1371/journal.pone.0104828

**Published:** 2014-10-03

**Authors:** François Montastruc, Guillaume Moulis, Aurore Palmaro, Virginie Gardette, Geneviève Durrieu, Jean-Louis Montastruc

**Affiliations:** 1 Laboratoire de Pharmacologie Médicale et Clinique, Faculté de Médecine de l'Université de Toulouse, Toulouse, France; 2 Service de Pharmacologie Médicale et Clinique, Centre Midi-Pyrénées de PharmacoVigilance, de Pharmacoépidémiologie et d'Informations sur le Médicament, Centre Hospitalier Universitaire, Toulouse, Toulouse, France; 3 INSERM U 1027, Université de Toulouse, Toulouse, France; 4 Département Universitaire d'Epidémiologie, d'Economie de la Santé et de Santé Publique, Faculté de Médecine de l'Université de Toulouse, Toulouse, France; 5 Service d'Epidémiologie, Centre Hospitalier Universitaire, Toulouse, France; 6 Service de Médecine Interne, Centre Hospitalier Universitaire, Toulouse, France; Royal College of Surgeons, Ireland

## Abstract

**Background:**

The present study aimed to describe exposure and attitudes of French medical residents towards pharmaceutical industry. The study was performed shortly after the Mediator affair which revealed several serious conflicts of interest inside the French health system.

**Methods and Findings:**

A cross-sectional study was implemented among residents from 6 French medical faculties. Independent education in pharmacology, attitudes towards the practices of pharmaceutical sales representatives, opinions concerning the pharmaceutical industry, quality of information provided by the pharmaceutical industry, and opinions about pharmaceutical company sponsorship were investigated through a web-based questionnaire. We also assessed potential changes in resident attitudes following the Mediator affair. The mean value of exposure to drug companies was 1.9 times per month. Global opinions towards drug company information were negative for 42.7% of the residents and positive for only 8.2%. Surprisingly, 81.6% of residents claimed that they had not changed their practices regarding drug information since the Mediator affair. Multivariate analyses found that residents in anesthesiology were less likely to be exposed than others (OR = 0.17 CI95% [0.05–0.61]), exposure was significantly higher at the beginning of residence (p<0.001) and residents who had a more positive opinion were more frequently exposed to drug companies (OR = 2.12 CI95% [1.07–4.22]).

**Conclusions:**

Resident exposure to drug companies is around 1 contact every 2 weeks. Global opinion towards drug information provided by pharmaceutical companies was negative for around 1 out of 2 residents. In contrast, residents tend to consider the influences of the Mediator affair on their practice as relatively low. This survey enabled us to identify profiles of residents who are obviously less exposed to pharmaceutical industry. Current regulatory provisions are not sufficient, indicating that further efforts are necessary to develop a culture of disclosure of conflict of interest and of transparency in residents.

## Introduction

Residence, a 3 to 5-year period for clinical practice in hospitals at the end of medical studies, is a critical time for young physicians to learn the principles that will guide them throughout their whole career, in particular prescribing habits. During this period, residents begin to interact with the pharmaceutical industry through information provided by pharmaceutical sales representatives (PSRs), at product meetings and/or in the medical literature they provide. Several studies have shown that drug companies tend to present informations on drugs which emphasize their benefits and underplay their risks [1]–[4], thus increasing the likelihood of their prescription [5]–[7]. Exposure to pharmaceutical representatives was found to be associated with irrational prescribing [8], [9] and additional cost [10] Relationships between drug companies and physicians often lead to gifts, sponsoring for diners or medical meetings [11]. In a survey among 3,167 U.S. physicians, Campbell reported that 94% of them had relationships with the pharmaceutical industry, most of them involving cocktails or diners in the workplace (83%). General practitioners met industry representatives more frequently than physicians from other specialties (internists, cardiologists, pediatricians, surgeons or anesthesiologists) [11].

Some North American but very few European surveys have studied residents' perceptions about interactions with drug-companies [12], [13]. These studies concluded that medical education provides substantial contact with pharmaceutical marketing, and that the extent of such contact is associated with both positive attitudes about marketing and skepticism about the negative implications of these interactions. However, little is known about individual factors associated with exposure to the pharmaceutical industry, in particular the influences of drug scandals.

In France, in 2011, the Mediator (benfluorex) affair raised the issue of health professionals' independence with regard to pharmaceutical industry [14]. Benfluorex, an amphetaminic derivative marketed by Servier under the brand name Mediator, was prescribed for 33 years as an adjunct for hyperlipidemia and diabetes but was also widely used off-label for obesity. This drug induced cardiac valvulopathy and pulmonary arterial hypertension and was estimated to have caused between 500 and 2,000 deaths [15]. Further investigations revealed some conflicts of interest with the pharmaceutical industry among assessors from the French medicines agency and prescribers [16]. This affair was widely covered by both French and international media and had an extensive influences on the public [14]–[17]. Following this public health crisis, it can be hypothesized that residents could have modified their opinions and attitudes about pharmaceutical information as well as their own practices.

Thus, the present study aimed to describe exposure and attitudes of French medical residents towards pharmaceutical industry.

## Methods

### Survey design

A Web-based questionnaire (Survey Questionnaire S1) was created for the purpose of the study, on the basis of 3 existing questionnaires from 2 North American [18], [19] and 1 previous European [20] studies. All items were extracted and examined for inclusion in the study questionnaire. Forty-seven items were retained, translated into French, and adapted to the French medical context. We added 7 other items focusing on the Mediator affair. All items were validated by author consensus after two rounds according to the Delphi Method [21]. The Delphi Method, developed by the Rand Corporation in the 1950s, is a research method allowing a consensus opinion to be reached among experts, using questionnaires, through an iterative process known as a round [22]. The responses from the first round were collected and analyzed. A revised questionnaire based on the results of this analysis was then submitted to the same experts. Finally, the final questionnaire comprised 54 questions, covering 7 main topics: 1-demographic information 2-academic education or any awareness-raising action concerning interactions with PSRs and conflict of interest, 3-attitudes toward PSRs, 4-opinions about the pharmaceutical industry, 5-perceived quality of information provided by the pharmaceutical industry, 6-opinions about pharmaceutical sponsorship and 7-potential changes in attitudes towards the pharmaceutical industry since the Mediator affair. In 27 out of 54 items, a 5-category Likert scale was used to assess residents' agreement with a series of statements related to the main topics (1 = strongly disagree, 2 = disagree, 3 = neutral, 4 = agree, and 5 = strongly agree) [23].

### Participants and data collection

This cross-sectional study was conducted among current medical residents of 6 French Medical faculties: the Joseph-Fournier University in Grenoble, Sophia Antipolis University in Nice, Mediterranean University in Marseille, University of Montpellier, Paul-Sabatier University in Toulouse, and Victor-Segalen University in Bordeaux. These 6 universities were selected because they were the only ones able to give us full access to all the E-mail addresses. They were representative of all the other French universities [24], [25]. E-mails inviting current residents to take part in the online survey were sent out from August 24th to October 2nd 2011. The E-mails specified that the work was being carried out for independent research purposes, supervised by Toulouse University. E-mail addresses were not directly communicated to the investigators (FM and others). No reminders were sent.

Medical residents did not receive any educational credits or financial compensation for taking part in the study. This survey was carried out in accordance with the principles outlined in the Declaration of Helsinki [26].

### Definition of exposure and measurement

Exposure to the pharmaceutical industry was defined as any contact with pharmaceutical companies during the current semester, including meeting(s) with pharmaceutical industry representatives and/or gift(s), conference(s), restaurant meal(s), thesis fees or book(s) paid for by a drug company. An exposure index (global and by type of exposure) was defined on the basis of the numbers of such contacts during the current semester. This index was obtained by dividing the number of contacts (without weighting), by the number of months, in order to interpret it as an average number of contacts per month.

### Statistical analysis

Survey responses were transferred to SAS 9.3 software (SAS Institute, Cary NC). Descriptive analyses used mean values ± standard deviation (SD) and proportions for quantitative and qualitative variables, respectively.

We first conducted a bivariate analysis using Pearson's χ2 test or Fisher's exact test for qualitative variables and Student's t-test or Mann Whitney parametric test for quantitative variables. A backward logistic regression model was built to assess the multivariate associations between residents' exposure index to the pharmaceutical industry per month and residents' particulars. The exposure index was dichotomized according to the median value, superior or inferior to 1.5. Independent variables associated with a p value under 0.25 in bivariate analysis and known confounding factors (gender and ad hoc training regarding conflict of interest), whatever their significance level, were included in the initial model. The following factors were therefore included in the multivariate model: gender, universities, medical specialties, education or any awareness-raising action about conflict of interest, opinion of the pharmaceutical industry, number of years in residence, change in their practices regarding drug information since the Mediator affair. Crude and adjusted Odds Ratios with their 95% confidence intervals were estimated and all P-values were two-tailed. The adequation of the final model was assessed using the Hosmer and Lemeshow test (alpha threshold: 5%).

## Results

### Study population

The response rate varied from 13.3% to 20.0% depending on the university [mean: 17.3% (631/3,642)]. Table 1 shows the main characteristics of the residents participating in the survey in each medical faculty. The residents were aged from 24 to 36 years (mean, 27.5 [1.9]) and 423 (68.0%) were women. Mean practice duration as resident was 1.65 years (1.20). In the final sample, 47.2% were residents in general practice, 20.3% medical specialist residents (cardiology, neurology, gastroenterology, internal medicine, dermatology, radiology, nephrology, pulmonology, oncology, hematology, endocrinology, rehabilitation, rheumatology, genetics), 9.8% surgery residents, 4.6% anesthesiology residents, 7.0% psychiatry residents and 11.1% residents in other specialties (pediatrics, gynecology, obstetrics, biology, public health). Among these 631 residents, 300 (48.0%) wished to follow an academic career and to be further involved in medical student education. The final sample did not differ significantly from the national proportion for gender, age and resident specialties [24], [25].

**Table 1:** Main characteristics of Residents (n = 631) from the different Medical Universities participating to the Survey.

Medical University	University Victor Segalen	University Paul Sabatier	University of Montpellier	University Méditerranée	University Nice Sophia Antipolis	University Joseph Fournier	Total
Location	Bordeaux	Toulouse	Montpellier	Marseille	Nice	Grenoble	-
Age mean (SD), years	27.8 (2.1)	27.5 (1.9)	27.4 (1.8)	27.3 (2.0)	26.9 (1.0)	27.4 (2.0)	27.5 (1.9)
Women, %	58.7	70.6	68.1	65.4	86.4	71.8	68.0
Response rate, Number (%)	93 (13.3)	156 (17.9)	122 (17.5)	134 (20.0)	22 (14.6)	104 (18.8)	631 (17.3)
Number of years in residence (%)							
0 yr	21 (22.6)	25 (16.0)	19 (15.6)	30 (22.4)	8 (36.5)	25 (24.0)	128 (20.3)
1 yr	18 (19.3)	38 (24.3)	37 (30.3)	42 (31.3)	4 (18.1)	25 (24.0)	164 (26.0)
2 yr	21 (22.6)	47 (30.1)	40 (32.8)	35 (26.1)	9 (40.9)	39 (37.6)	191 (30.3)
3 yr	22 (23.6)	33 (21.1)	19 (15.6)	14 (10.4)	1 (4.5)	9 (8.6)	98 (15.5)
4 yr	11 (11.8)	12 (7.7)	7 (5.7)	12 (8.9)	0	6 (5.8)	48 (7.6)
5 yr	0	1 (0.6)	0	1 (0.7)	0	0	2 (0.3)
Specialty (number, %)							
Family practice	21 (22.6)	71 (45.5)	57 (46.7)	66 (49.3)	22 (100)*	61 (58.7)	298 (47.2)
Medical specialty†	27 (29.0)	29 (18.5)	26 (21.3)	24 (17.9)	0	22 (21.2)	128 (20.3)
Surgery	17 (18.2)	14 (9.0)	13 (10.7)	12 (9.0)	0	6 (5.8)	62 (9.8)
Anesthesiology	5 (5.4)	9 (5.8)	5 (4.1)	8 (6.0)	0	2 (1.9)	29 (4.6)
Psychiatry	5 (5.4)	16 (10.3)	10 (8.2)	8 (6.0)	0	5 (4.7)	44 (7.0)
Others‡	18 (19.4)	17 (10.9)	11 (9.0)	16 (11.8)	0	8 (7.7)	70 (11.1)

* In Nice University, only residents in family practice were included.

† Medical specialty: Cardiology, Neurology, Gastroenterology, Internal Medicine, Dermatology, Radiology, Nephrology, Pulmonology, Oncology, Hematology, Endocrinology, Physical Medicine and Rehabilitation, Rheumatology, Genetic.

‡ Others: Pediatry, Gynecology, Obstetric, Public health, Biology.

Abbreviations SD: Standard Deviation; yr: year.

### Awareness-raising action with regard to conflict of interest

Only 1 out of 3 (30%; n = 189) residents claimed that they had previously received any education or undergone an awareness-raising program concerning conflict of interest with regard to drug companies. Only 17% (n = 110) had received advice from their university hospitals. Most of the residents (67%; n = 419) expected their university hospital to give lectures about interactions with the pharmaceutical industry and 72% (n = 454) claimed that their training with regard to conflict of interest was unsatisfactory.

### Exposure

Types of exposure and the corresponding index are detailed in Table 2. A total of 551 residents (87.2%) responded to all exposure items. Mean value of exposure index was 1.9 per month (SD, 1.4; range, 0–9.3). Thus, each student attended approximately one sponsored activity or received one gift every 2 weeks. Most of the residents (95.4%) had already received at least one PSR visit (mean value: 1.34 times monthly, SD, 1.04; range, 0–3.5) and 85.0% of the residents had previously been invited to a restaurant by a drug company (mean value: 0.24 times monthly, SD, 0.31; range, 0–3.5).

**Table 2:** Type of Residents' Exposure with Drug Companies.

Type of Event or Gift	Residents, No (n = 631)	Residents Who participated in or received a gift ≥1 Event, n (%)	Exposure Frequency per Month	
			Mean (SD)	Range
Meetings with pharmaceutical sale representative	629	600 (95.4)	1.34 (1.04)	0–3.50
Gift ≥50 €	585	71 (12.1)	0.03 (0.11)	0–1.16
Gift <50 €	590	356 (60.3)	0.19 (0.46)	0–5.30
Congress or conference paid by a drug company	599	215 (35.9)	0.06 (0.14)	0–1.0
Restaurant provided by a drug company	622	529 (85.0)	0.24 (0.31)	0–3.5
Thesis paid by a drug company	580	40 (6.8)	0.01 (0.06)	0–1.0
Book donated by a drug company	593	102 (5.2)	0.03 (0.09)	0–1.0

Abbreviations SD: Standard Deviation.

### Opinions

Globally, opinions with regard to drug company information were negative for 42.7% (n = 266) of the residents, positive for 8.2% (n = 55) and neutral for 49.1% (n = 310). There was no significant difference between the different medical faculties (p = 0.752). About 1 out of 2 residents thought it was acceptable that the pharmaceutical industry should organize medical meetings or conferences for students but most of them (60.6%, n = 381) did not want lectures planned by pharmaceutical companies to be organized inside the hospital. A large number of residents (n = 553, 88.2%) wanted lecturers to disclose any conflict of interest with the industry.

### Drug company information (Table 3)

**Table 3:** Information from drug companies.

Statement	Strongly disagree, n (%)	Disagree, n (%)	Neutral, n(%)	Agree, n (%)	Strongly agree, n (%)
Information from pharmaceutical industry is of good quality (n = 625)	90 (14.4)	226 (36.2)	231 (37.0)	74 (11.8)	4 (0.6)
Information from pharmaceutical industry is valuable to me (n = 625)	125 (20.0)	197 (31.5)	159 (25.5)	122 (19.5)	22 (3.5)
Drug company-sponsored grand rounds are often biased in favor of the company's products (n = 626)	18 (2.9)	65 (10.3)	78 (12.5)	233 (37.2)	232 (37.1)
Your knowledge is sufficient to be not influenced by pharmaceutical industry (n = 624)	51 (8.2)	169 (27.1)	183 (29.3)	168 (26.9)	53 (8.49)
Advertising for prescription-only drugs in medical journals should not be allowed (n = 623)	27 (4.3)	92 (14.8)	141 (22.6)	117 (18.8)	246 (39.5)

Half of the residents felt that the quality of information provided by the industry was not good (50.6%; n = 316) and not of value to them (51.5%; n = 322). Other responses suggested skepticism about drug company marketing. Three quarters of the residents (74.3%; n = 465) thought that industry information was biased in favor of their products. Fifty eight percent (n = 363) wanted advertising for prescription-only drugs to be banned from medical journals.

### Pharmaceutical sponsorship (Table 4)

**Table 4:** Pharmaceutical financial sponsoring.

Statement	Strongly disagree, n (%)	Disagree, n (%)	Neutral, n (%)	Agree, n (%)	Strongly agree, n (%)
It is acceptable that a medical Professor receive money from drug companies for medical congress (n = 624)	143 (22.9)	110 (17.6)	152 (24.4)	148 (23.7)	71 (11.4)
Since students from non-medical universities are invited to meetings and meals by private companies, medical residents should be allowed to attend similar arrangements with pharmaceutical industry (n = 627)	79 (12.6)	72 (11.5)	166 (26.5)	135 (21.5)	175 (27.9)
It is acceptable that medical residents receive financial sponsoring from drug companies, because they haven't enough money to be informed (n = 625)	162 (25.9)	92 (14.7)	107 (17.1)	144 (23.1)	120 (19.2)
It is acceptable that medical residents receive financial sponsoring from drug companies, because drug companies have minimal influence on residents (n = 626)	199 (31.8)	156 (24.9)	109 (17.4)	112 (17.9)	50 (8.0)

Concerning funding by pharmaceutical companies, opinions of the residents were ambivalent. Indeed, 40.5% of the residents (n = 253) did not approve of pharmaceutical companies paying a professor or a senior for giving a talk at a conference, but half of them (49.4%; n = 310) agreed that receiving invitations to conferences from pharmaceutical companies was acceptable. In contrast, a majority (56.7%; n = 355) believed that it is unacceptable for medical residents to receive financial sponsorship from drug companies.

### Influence of the Mediator affair

Since the Mediator affair, most of the residents (509, 81.6%) claimed that they had not changed their practices regarding information on drugs. To obtain information concerning drugs, residents claimed they use the French drug dictionary Vidal as a primary source (n = 338, 53.9%), followed by independent medical journals (n = 108, 17.2%), national health agency [Haute Autorité de Santé (HAS) and/or Agence Nationale de Sécurité du Médicament (ANSM)] websites (n = 90, 14.4%), scientific medical journals (n = 51, 8.1%), university education (n = 14, 2.3%), Internet resources (Google, Yahoo) (n = 10, 1.6%) or other means (PSRs) (n = 10, 1.6%). Seventy-five per cent of the residents claimed that drug assessment experts working in regulatory agencies may be biased due to links with the pharmaceutical industry. Residents would want experts to publish their links with the industry (91.0%). In case of conflict of interest, residents also wanted experts to not be permitted to give their opinion on drugs (67.0%). In contrast, only 11.0% (n = 69 residents) claimed to consult conflict of interest statements when reading a medical article. Finally, only 132 residents (21.0%) agreed with prohibition of any PSR visits to hospitals.

### Factors associated with medical resident exposure (Table 5)

Table 5 shows the results of bivariate and multivariate analyses. Multivariate analyses found that the exposure index significantly differed according to specialty, number of years in residence and opinion concerning the pharmaceutical industry.

**Table 5:** Predictors of Resident-Industry Relationships.

Characteristics	Exposure ≥1.5 per month				
	Percentage (%)	Bivariate analysis		Multivariate analysis Final Model* (n = 543)	
		OR [CI 95%]	p-value	OR [CI 95%]	p-value
**Gender (n = 543)**					
Male (n = 172)	55.23	1	0.352		
Female (n = 371)	50.94	0.84 [0.58–1.21]			
**University (n = 551)**					
Marseille (n = 115)	51.30	1	0.031		
Bordeaux (n = 85)	56.47	1.23 [0.70–2.16]			
Toulouse (n = 132)	44.70	0.77 [0.46–1.27]			
Montpellier (n = 108)	54.63	1.14 [0.67–1.93]			
Nice (n = 20)	90.00	8.54 [1.89–38.51]			
Grenoble (n = 91)	48.35	0.89 [0.51–1.54]			
**Specialty (n = 551)**					
Family practice (n = 260)	50.77	1	<0.001	1	<0.001
Medical specialty† (n = 109)	66.97	1.97 [1.23–3.14]		1.69 [0.99–2.86]	
Surgery (n = 56)	64.29	1.75 [0.96–3.18]		1.86 [0.96–3.58]	
Anesthesiology (n = 24)	12.50	0.14 [0.04–0.48]		0.17 [0.05–0.61]	
Psychiatry (n = 42)	35.71	0.54 [0.27–1.06]		0.59 [0.29–1.21]	
Others‡ (n = 60)	46.67	0.85 [0.48–1.49]		0.87 [0.48–1.58]	
**Lectures about conflicts of interest during student medical courses (n = 551)**					
No (n = 392)	53.32	1	0.364		
Yes (n = 159)	49.06	0.84 [0.58–1.22]			
**Interested in pursuing an academic career (n = 546)**					
No (n = 280)	51.43	1	0.721		
Yes (n = 266)	53.01	1.06 [0.76–1.49]			
**Opinion about pharmaceutical industry (n = 551)**					
Negative-neutral (n = 502)	50.20	1	0.006	1	0.032
Positive (n = 49)	71.43	2.48 [1.30–4.72]		2.12 [1.07–4.22]	
**Subscription independent medical journal (n = 548)**					
No (n = 356)	51.97	1	0.886		
Yes (n = 192)	52.60	1.03 [0.72–1.46]			
**Change in my practice about drug information (n = 544)**					
No (n = 448)	51.79	1	0.542		
Yes (n = 96)	55.21	1.15 [0.74–1.79]			
**Number of years in residence (n = 551)**					
0 year (n = 114)	76.32	1	<0.001	1	<0.001
1 years (n = 134)	52.99	0.35 [0.20–0.61]		0.38 [0.21–0.67]	
2 years (n = 175)	37.71	0.19 [0.11–0.32]		0.20 [0.11–0.34]	
≥3 years (n = 128)	49.22	0.30 [0.17–0.52]		0.25 [0.14–0.46]	

† Medical specialty: Cardiology, Neurology, Gastroenterology, Internal Medicine, Dermatology, Radiology, Nephrology, Pulmonology, Oncology, Hematology, Endocrinology, Physical Medicine and Rehabilitation, Rheumatology, Genetic.

‡ Others: Pediatry, Gynecology, Obstetric, Public health, Biology.

* The following factors were therefore included in the model: gender, universities, medical specialties, education or any awarenessraising action about conflicts of interest, opinion about pharmaceutical industry, number of years in residence, change in their practice about drug information since Mediator's affair.

Abbreviations OR: Odds Ratio; CI 95%: Confidence Interval 95%.

Only residents in anesthesiology were less likely to be exposed than other residents (OR = 0.17 CI95% [0.05–0.61]). Exposure was significantly higher at the beginning of residence (p<0.001). Residents who had a fairly positive opinion concerning the pharmaceutical industry were exposed to drug companies twice as much (OR = 2.12 CI95% [1.07–4.22]).

Adjusted analysis did not demonstrate any association with other variables studied: gender, university, changes in practice with regard to drug information since the Mediator affair and raising of awareness concerning conflict of interest during student medical courses.

## Discussion

Our descriptive multicenter study provides new evidence on factors associated with interactions between residents and drug companies following the Mediator affair. In our sample, we found that resident-industry relationships are common in medical practice (contact around twice a month). Furthermore, our model suggests that exposure of residents to drug companies varies according to a resident's particulars, opinions or specialties: older residents, residents with negative or neutral opinions concerning the pharmaceutical industry or residents in anesthesiology were less exposed to drug companies.

The most interesting result is that the mean value of exposure to drug companies was 1.9 times per month. Previous studies [27]–[29] found higher values (between 2 and 10 contacts per month). However, these works did not use the same criteria index and were carried out in different contexts, mainly with young medical students (and not residents who are older and are finishing their studies with clinical practice in university hospitals). Sierles [28] assessed exposure of U.S. medical students without taking into account PSR visits. Our index exposure was more appropriate to estimate all contacts (PSR visits, gifts, restaurants, etc.) between residents and drug companies. Concerning lunches paid by pharmaceutical companies, we found a frequency of exposed residents (around 85%) similar to previous studies. Bellin [29] underlined that clinical students are easily attracted by lunch and gifts of little value. In the medical field, it is commonly thought that small gifts do not influence practice. However, several studies have shown that accepting a gift may lead to feelings of indebtedness and therefore reduces critical thinking [30]–[32].

The second main result concerns opinions about the pharmaceutical industry. In fact, overall opinions towards drug company information were negative for about half of the residents and three quarters of them thought that industry information was biased in favor of their products. In contrast, they found that medical meetings or conferences supported by pharmaceutical companies could be of interest. Thus, attitudes of residents about the pharmaceutical industry appear to be ambivalent. As far as we know, there are very few studies on this topic of opinions of medical students with regard to drug companies. Lea [20] found values similar to our survey.

The third result is surprising. Despite the Mediator affair in terms of public health [17], more than 80% of the residents claimed that they had not changed their practices regarding drug information. They mainly used official data from health authorities (the French drug dictionary Vidal, French medicines agency) more than industry resources as a primary source of information. Finally, despite their skepticism about the reliability of drug assessment by national health agencies, residents tend to consider the influences of the Mediator affair on their practice as relatively low. Once again, behavior of residents concerning drug information is ambivalent.

Since the survey period, the economic environment due to financial crisis has affected pharmaceutical industry in Europe. As a result of budget constraints, the number of sales representatives has been reduced. Thus, current number of contacts per residents may not be as high as reported in our survey.

Our study has several limitations. Firstly, given that the data was declarative, the potential for a bias of social desirability [33], whereby residents might have tended to minimize their exposure to the pharmaceutical industry, might be suggested. However, this bias is probably less marked in our study because the questionnaire was conducted via Internet [34] and was anonymous. Secondly, since our survey was designed as a cross-sectional study, we were unable to investigate the consequences of the Mediator affair. Questions regarding past experiences during residency might have induced a memorization bias, even though residents were questioned over a relatively short period. Finally, our study is only descriptive.

In contrast, our work has several strengths. First, our response rate (between 13.3% and 20.0%) is relatively high for such a study, taking into account that the questionnaire was sent only once and via the Internet [35]. The study sample was representative of the main characteristics of the French medical resident population in general (gender, age, specialties). In comparison with many other surveys (see the systematic review [12]), the number of residents included in our study is high (n = 631). Second, the present work is the first to also investigate factors associated with resident exposure by multivariate logistic regression. This survey enabled us to identify profiles of residents who are obviously less exposed to pharmaceutical industry: older residents (i.e. after the first year of residence), residents with negative or neutral opinion concerning the pharmaceutical industry or residents in anesthesiology. In contrast, residents in medical specialties or surgery seemed to be more exposed than those in general practice. The findings also tend to indicate that current French regulatory provisions regarding PSRs are not sufficient to limit the exposure of young doctors to the pharmaceutical industry [36].

This multicenter survey of a large population of residents from six different universities raises some important issues. Very few residents have been educated during their pre-clinical training with regard to conflict of interest with the pharmaceutical industry. Unlike American associations [37], French universities and resident associations did not seem aware of this issue. This survey highlights the fact that residents would like to receive training and an ethical framework concerning medical-industry relationships from their hospital university, as is already the case in U.S. medical schools. [37]–[38] Residents expressed their need for more independent information about drugs, but also for training. They would like to be given guidelines detailing good practices for interaction with the pharmaceutical industry from their university. Further efforts are still necessary to develop a culture of disclosure of conflict of interest and of transparency in residents, and to provide intensive academic pharmacological information during residency.

In conclusion, this study shows that medical resident exposure to drug companies is around 1 contact every 2 weeks. Global opinion towards drug information provided by pharmaceutical companies was negative for around 1 out of 2 residents. Moreover, there are relatively few changes in their practice following the Mediator affair. This survey enabled us to identify associated factors to exposure to pharmaceutical industry. Current regulatory provisions are not sufficient, indicating that further efforts are necessary to develop a culture of disclosure of conflict of interest and of transparency in residents.

## Supporting Information

Survey Questionnaire S1 (DOC)

## References

[1] Cho MK, Bero LA (1996) The quality of drug studies published in symposium proceedings. Ann Intern 124: 485–89. 10.7326/0003-4819-124-5-199603010-00004 8602706

[2] Lexchin J, Bero LA, Djulbegovic B, Clark O (2003) Pharmaceutical industry sponsorship and research outcome and quality: systematic review. BMJ 326: 1167–70. 12775614 10.1136/bmj.326.7400.1167 PMC156458

[3] Villanueva P, Peiró S, Librero J, Pereiró I (2003) Accuracy of pharmaceutical advertisements in medical journals. Lancet 361: 27–32. 12517463 10.1016/S0140-6736(03)12118-6

[4] Mintzes B, Lexchin J, Sutherland JM, Beaulieu M-D, Wilkes MS, et al (2013) Pharmaceutical Sales Representatives and Patient Safety: A Comparative Prospective Study of Information Quality in Canada, France and the United States. J Gen Intern 28: 1368–75. 10.1007/s11606-013-2411-7 PMC3785667 23558775

[5] Bowman MA, Pearle DL (1988) Changes in drug prescribing patterns related to commercial company funding of continuing medical education. J Contin Educ Health Prof 8: 13–20. 10294441 10.1002/chp.4750080104

[6] Orlowski JP, Wateska L (1992) The effects of pharmaceutical firm enticements on physician prescribing patterns. There's no such thing as a free lunch. Chest 102: 270–73. 1623766 10.1378/chest.102.1.270

[7] Prosser H, Almond S, Walley T (2003) Influences on GPs' decision to prescribe new drugs-the importance of who says what. Fam Pract 20: 61–68. 12509373 10.1093/fampra/20.1.61

[8] Wazana A (2000) Physicians and the pharmaceutical industry: is a gift ever just a gift? JAMA 283: 373–80. 10647801 10.1001/jama.283.3.373

[9] Halperin EC, Hutchison P, Barrier RC Jr (2004) A population-based study of the prevalence and influence of gifts to radiation oncologists from pharmaceutical companies and medical equipment manufacturers. Int J Radiat Oncol Biol Phys 59: 1477–83. 15275735 10.1016/j.ijrobp.2004.01.052

[10] Caudill TS, Johnson MS, Rich EC, McKinney WP (1996) Physicians, pharmaceutical sales representatives, and the cost of prescribing. Arch Fam Med 5: 201–6. 8769907 10.1001/archfami.5.4.201

[11] Campbell EG, Gruen RL, Mountford J, Miller LG, Cleary PD, et al (2007) A national survey of physician-industry relationships. N Engl J Med 356: 1742–50. 17460228 10.1056/NEJMsa064508

[12] Austad KE, Avorn J, Kesselheim AS (2011) Medical students' exposure to and attitudes about the pharmaceutical industry: a systematic review. PLoS Med 8: 1001037. 10.1371/journal.pmed.1001037 PMC3101205 21629685

[13] Lea D, Spigset O, Slørdal L (2010) Norwegian medical students' attitudes towards the pharmaceutical industry. Eur J Clin Pharmacol 66: 727–33. 20300742 10.1007/s00228-010-0805-6

[14] Mullard A (2011) Mediator scandal rocks French medical community. Lancet 377: 890–92. 21409784 10.1016/s0140-6736(11)60334-6

[15] Fournier A, Zureik M (2012) Estimate of deaths due to valvular insufficiency attributable to the use of benfluorex in France. Pharmacoepidemiol Drug Saf 21: 343–51. 22318872 10.1002/pds.3213

[16] Menard J (2011) Benfluorex: analysis of a drug-related public health crisis. Diabetes Metab 37: 169–75. 21612964 10.1016/j.diabet.2011.04.001

[17] Abou Taam M1, Rossard C, Cantaloube L, Bagheri H, et al (2014) Analysis of patients' narratives posted on social media websites on benfluorex's (Mediator) withdrawal in France. J Clin Pharm Ther 39: 53–5. 24304185 10.1111/jcpt.12103

[18] McKinney WP, Schiedermayer DL, Lurie N, Simpson DE, Goodman JL, et al (1990) Attitudes of internal medicine faculty and residents toward professional interaction with pharmaceutical sales representatives. JAMA 264: 1693–97. 2398609

[19] Sierles FS, Brodkey AC, Cleary LM, McCurdy FA, Mintz M, et al (2005) Medical students' exposure to and attitudes about drug company interactions: A national survey. JAMA 294: 1034–42. 16145023 10.1001/jama.294.9.1034

[20] Lea D, Spigset O, Slørdal L (2010) Norwegian medical students' attitudes towards the pharmaceutical industry. Eur J Clin Pharmacol 66: 727–33. 20300742 10.1007/s00228-010-0805-6

[21] Jones J, Hunter D (1995) Consensus methods for medical and health services research. BMJ 311: 376–80. 7640549 10.1136/bmj.311.7001.376 PMC2550437

[22] Dalkey NC (1969) The Delphi Method. Available: http://www.rand.org/pubs/research_memoranda/RM5888.html. Accessed 2013 Jul.

[23] Likert R (1932) A technique for the measurement of attitude. Archives of Psychology 140: 55.

[24] Fauvet L (2010) Les affectations des étudiants en médecine à l'issue des épreuves classantes nationales en 2010. Available: http://www.epsilon.insee.fr/jspui/handle/1/12491. Accessed 2013.

[25] Ministère des Affaires sociales et de la Santé (2011) Rapport 2010–2011 - Ministère des Affaires sociales et de la Santé. Available: http://www.sante.gouv.fr/rapport-2010-2011.html. Accessed 2013.

[26] WMA Declaration of Helsinki (2008) Ethical Principles for Medical Research Involving Human Subjects. Available: http://www.wma.net/en/30publications/10policies/b3/index.html. Accessed 2013.

[27] Monaghan MS, Galt KA, Turner PD, Houghton BL, Rich EC, et al (2003) Student understanding of the relationship between the health professions and the pharmaceutical industry. Teach Learn Med 15: 14–20. 12632703 10.1207/S15328015TLM1501_04

[28] Sierles FS, Brodkey AC, Cleary LM, McCurdy FA, Mintz M, et al (2005) Medical students' exposure to and attitudes about drug company interactions: a national survey. JAMA 294: 1034–42. 16145023 10.1001/jama.294.9.1034

[29] Bellin M, McCarthy S, Drevlow L, Pierach C (2004) Medical students' exposure to pharmaceutical industry marketing: a survey at one U.S. medical school. Acad Med 79: 1041–45. 15504768 10.1097/00001888-200411000-00005

[30] Dana J1, Loewenstein G (2003) A social science perspective on gifts to physicians from industry. JAMA 290: 252–5. 12851281 10.1001/jama.290.2.252

[31] Fabbri A, Ardigo M, Grandori L, Reali C, Bodini C, et al (2008) Conflicts of interest between physicians and the pharmaceutical industry. A quali-quantitative study to assess medical students' attitudes at the University of Bologna. Ricerca Sul Campo 24: 242–54.

[32] Sandberg WS, Carlos R, Sandberg EH, Roizen MF (1997) The effect of educational gifts from pharmaceutical firms on medical students' recall of company names or products. Acad Med 72: 916–18. 9347716

[33] Crowne DP, Marlowe D (1960) A new scale of social desirability independent of psychopathology. J Consult Psychol 24: 349–54. 13813058 10.1037/h0047358

[34] Heerwegh D, Loosveldt G (2008) Face-to-Face versus Web Surveying in a High-Internet-Coverage Population Differences in Response Quality. Public Opin Q 72: 836–46.

[35] Edwards PJ, Roberts I, Clarke MJ, Diguiseppi C, Wentz R, et al (2009) Methods to increase response to postal and electronic questionnaires. Cochrane Database Syst Rev 10.1002/14651858.MR000008.pub4 PMC8941848 19588449

[36] Ministère des Affaires sociales et de la Santé (2010) La charte de la visite médicale. Available: http://www.sante.gouv.fr/la-charte-de-la-visite-medicale.html. Accessed 2013.

[37] American Medical Student (2014) Association Conflict of Interest Policies at Academic Medical Centers. Available: http://www.amsascorecard.org/. Accessed 2014.

[38] Vuorenkoski L, Valta M, Helve O (2008) Effect of legislative changes in drug promotion on medical students: questionnaire survey. Med Educ 42: 1172–1177. 19120947 10.1111/j.1365-2923.2008.03169.x

