# Supplementary material for: Interactions between Medical Residents and Drug Companies: A National Survey after the Mediator® Affair
Source: PLoS One. 2014 Oct 3;9(10):e104828. doi: 10.1371/journal.pone.0104828 (PMC4184806; doi:10.1371/journal.pone.0104828)
Supplement: Survey Questionnaire S1 — (DOC) [file pone.0104828.s001.doc]

**Questionnaire Industrie pharmaceutique et Internes en médecine**

Dans le cadre d'un travail de recherche effectué au CHU de Toulouse, nous interrogeons les Internes en Médecine sur leur relation avec l’Industrie Pharmaceutique.

Ce questionnaire est entièrement anonyme. Il prendra *n* minutes de votre temps.

Merci de répondre le plus spontanément possible et de noter qu'il est très important de bien répondre aux questions dans l'ordre et de ne pas revenir aux questions précédentes.

Nous vous remercions par avance pour votre participation.

Nous insistons sur l’anonymat du questionnaire, vous pouvez donc répondre en pleine confiance.

Vous pouvez nous contacter par email pour des informations complémentaires.

[francoismontastruc@hotmail.fr](mailto:francoismontastruc@hotmail.fr)

- Je suis interne, j'ai compris les explications concernant cette étude et je consens à y participer * (obligatoire)

Oui/non

**A. Questions sociodémographiques**

1. année de naissance

1978 ou avant /1979/1980/…/1989 ou après

1. Sexe

Masculin/ féminin

1. CHU d’appartenance pour l’internat

BORDEAUX/GRENOBLE/MONTPELLIER/MARSEILLE/NICE/TOULOUSE/

1. Région d’externat (Faculté d’origine)

PARIS/BORDEAUX/LYON/MARSEILLE…/TOULOUSE/TOURS

1. Nombre de semestres validés

0 à 12

1. Filière

Médecine Générale/ Médecine Spécialisée/ Chirurgie/ Anesthésie réanimation/…/Santé publique

1. Spécialité de DES (uniquement pour la chirurgie et les spécialités médicales)

Chirurgie générale/Neurochirurgie/Ophtalmologie/ORL/stomatologie/anatomopathologie/Cardiologie/Dermatologie/Endocrinologie/Gastroenterologie/Genetique/Hematologie/Médecine interne/Médecine Nucleaire/MPR/Nephrologie/Neurologie/oncologie/Pneumologie/Radiologie/Rhumathologie

1. Pensez-vous bénéficier d’un poste de chef de clinique (CCA) ou d’assistant hospitalo-universitaire (AHU) après votre internat ? oui/non

**B. Formation**

1. J’ai choisi la carrière de médecin (vous pouvez sélectionner plusieurs propositions)

-Pour avoir un haut niveau social

-Pour avoir un travail intéressant

-Pour gagner de l’argent

-Pour aider les autres

-autre

1. Au cours de mon externat, j’ai reçu une formation (cours) sur les conflits d’intérêts avec l’industrie et l’information qu’elle procure

Oui/non

1. Avez-vous reçu des recommandations venant de votre hôpital ou de votre université à propos de la conduite à tenir vis-à-vis des représentants de l’industrie pharmaceutique ?

oui/pas à ma connaissance

1. L’hôpital devrait organiser des réunions d’information sur comment se comporter avec les représentants de l’industrie pharmaceutique.

Oui/ non

1. Vous estimez que votre formation sur les conflits d’intérêts est:

Très insuffisante 1 /…/ Très satisfaisante 5 (échelle likert)

1. Vous estimez que votre formation doit nécessairement se faire en lien avec l’industrie pharmaceutique

1/…/5

**C. Visite Médicale et exposition**

1. Combien de fois avez-vous rencontré un représentant de l’industrie pharmaceutique (visiteur (se) médical (e)) au cours des 6 derniers mois à l’hôpital ?

-Jamais

-de 1 à 5 fois

-de 6 à 10 fois

-de 11 à 20 fois

- > 20 fois

Au sujet des visiteurs médicaux à l’hôpital

1. Je les reçois quand ils se présentent oui, toujours/oui, parfois/non, jamais
2. Je les reçois uniquement sur rendez-vous oui, toujours/oui, parfois/non, jamais
3. Je ne les reçois jamais oui/non

Quand je rencontre un visiteur médical

1. Je demande le RCP « résumé des caractéristiques du produit » toujours/parfois/jamais
2. Je demande l’avis de la CT « commission de transparence » toujours/parfois/jamais

Depuis le début de votre internat, combien de fois avez-vous reçu un cadeau de l’industrie pharmaceutique

1. D’une valeur supérieure à 50 euros 0/ 1-4/5-9/10-15/>15/ je ne sais pas
2. D’une valeur inférieure à 50 euros 0/ 1-4/5-9/10-15/>15/ je ne sais pas
3. Pour payer des frais de cours ou de congrès 0/ 1-4/5-9/10-15/>15/ je ne sais pas
4. Pour payer un repas ou une soirée 0/ 1-4/5-9/10-15/>15/ je ne sais pas
5. Pour payer la thèse (frais d’impression, apéro, soirée…) 0/ 1-4/5-9/10-15/>15/ je ne sais pas
6. Pour payer des bouquins 0/ 1-4/5-9/10-15/>15/ je ne sais pas

**D. Votre opinion générale**

**Merci de bien vouloir indiquer pour chacune des propositions à quel niveau vous êtes d’accord**

1. Si vous deviez caractériser votre opinion vis-à-vis de l’industrie pharmaceutique, vous diriez qu’elle est :

1 (très positive) / 2 / 3 / 4 / 5 (très négative)

1. Les rapports entre les médecins et l’industrie pharmaceutique n’est pas bénéfique pour les patients

1/…/5

1. Il est acceptable que l’industrie pharmaceutique organise des réunions médicales, des congrès pour les internes en médecine

1(pas d’accord)/2/…/5 (tout à fait d’accord)

1. L’industrie pharmaceutique ne devrait pas être autorisée à organiser des réunions médicales, des staffs à l’hôpital

1/…/5

1. C’est pertinent lorsqu’avant une réunion médicale l’intervenant déclare ses possibles liens d’intérêts

1/…/5

1. Il est acceptable de donner aux patients des documents d’information ou d’éducation faits par des laboratoires pharmaceutiques

1/…/5

1. L’industrie dépense trop d’argent pour le marketing de ses produits avec les médecins

1/…/5

**E. Information et Financement**

**Merci de bien vouloir indiquer pour chacune des propositions à quel niveau vous êtes d’accord**

1. L’information sur le médicament de l’industrie pharmaceutique est de bonne qualité

1/…/5

1. L’information de l’industrie pharmaceutique est importante pour ma formation.

1/…/5

1. Une information indépendante sur le médicament garantit une information de meilleure qualité

1/ …/5

1. Les staffs/ réunions sponsorisés par l’industrie pharmaceutique sont souvent biaisé en faveur des produits de la compagnie

1/…/5

1. Je pense avoir suffisamment de connaissance pour ne pas être influencé par l’industrie pharmaceutique

1/…/5

1. Les informations provenant de l’industrie pharmaceutique peuvent affecter mes habitudes de prescription

De façon positive/ de façon négative / n’affecte pas mes habitudes de prescriptions

1. L’industrie pharmaceutique omet de nombreuses informations lors de la présentation de leurs médicaments

1/…/5

1. La publicité sur les médicaments de prescription ne doit pas être autorisée dans les journaux médicaux.

1/…/5

1. Je suis abonné à une revue médicale indépendante (Prescrire…) oui/non
2. Dans la mesure où d’autres professionnels non médecins sont invités par des compagnies privée à des repas ou congrès, les internes en médecines doivent bénéficier des mêmes droits

1/…/5

1. Je ne vois pas comme un problème le fait qu’un professeur de médecine reçoive de l’argent de l’industrie pharmaceutique
   1. Pour des symposiums/séminaires
   2. Pour des congrès
   3. Pour des travaux de recherche
2. Je pense que l’influence d’un message commercial est plus forte lorsqu’il s’accompagne d’un cadeau.

1/…/5

1. Il est acceptable que les internes reçoivent des financements de l’industrie pharmaceutique parce qu’ils n’ont pas assez d’argent pour se former

1/…/5

1. Il est acceptable que les internes reçoivent des financements de l’industrie pharmaceutique parce qu’elle a peu d’influence sur eux

1/…/5

**F. Depuis l’affaire du Benfluorex « MEDIATOR ®»…**

1. Depuis l’affaire du « MEDIATOR », je pense qu’on devrait interdire les visiteurs médicaux pour ne pas influencer la prescription médicale
2. Depuis l’affaire du « MEDIATOR », j’ai modifié ma pratique pour m’informer sur le médicament
3. Depuis l’affaire du « MEDIATOR », je m’informe en priorité sur le médicament auprès (1 seule réponse possible):
   1. Des visiteurs médicaux
   2. Du Vidal
   3. Pendant les cours de DES
   4. Des Agences Nationales (HAS, AFSSaPS…)
   5. Des services de Pharmacologie ou des centres régionaux de PharmacoVigilance
   6. Dans les revues scientifiques (articles originaux indexés)
   7. A partir des sources de données indépendantes (Revue Prescrire, Bulletin de PharmacoVigilance)
   8. Sur internet, à partir d’un moteur de recherche (google, yahoo…)
   9. Autres
4. Depuis l’affaire du « MEDIATOR », lorsque je lis un article, je recherche systématiquement les conflits d’intérêts des auteurs.

Oui, maintenant je le fais/ Oui, mais je le faisait avant / Non, je ne le fais pas

1. Je pense que les évaluateurs du médicament peuvent avoir une expertise biaisée du fait de liens avec l’industrie pharmaceutique

1/…/5

1. Je pense que les évaluateurs du médicament doivent publier leurs liens avec des industries pharmaceutiques

1/…/5

1. Je pense que les évaluateurs du médicament ne doivent pas donner leur avis en cas de liens avec des industries pharmaceutiques

1/…/5
